# CLEC4M is associated with poor prognosis and promotes cisplatin resistance in NSCLC patients

**DOI:** 10.7150/jca.30139

**Published:** 2019-10-19

**Authors:** Li-Ming Tan, Xi Li, Cheng-Feng Qiu, Tao Zhu, Cheng-Ping Hu, Ji-Ye Yin, Wei Zhang, Hong-Hao Zhou, Zhao-Qian Liu

**Affiliations:** 1 Department of Clinical Pharmacology, Xiangya Hospital, Central South University, Changsha 410008, P. R. China;; 2 Department of Evidence-based Medicine and Clinical Center, The First People's Hospital of Huaihua City,Huaihua 418000, P. R China;; 3 Institute of Clinical Pharmacology, Central South University, Hunan Key Laboratory of Pharmacogenetics, Changsha 410078, P. R. China;; 4 National Clinical Research Center for Geriatric Disorders, Changsha 410008, P.R. China;; 5 Department of Respiratory Medicine, Xiangya Hospital, Central South University, Changsha, Hunan 410008, P. R. China;; 6 Department of Clinical Pharmacy, The Second People's Hospital of Huaihua City, Huaihua 418000, P. R China.

**Keywords:** CLEC4M, non-small cell lung cancer, cisplatin resistance

## Abstract

Cisplatin-based chemotherapy is the foundation of treatment for major non-small cell lung cancer (NSCLC) patients. However, cisplatin resistance is still a challenging issue, and the molecular mechanisms underlying this resistance remain to be fully explored. CLEC4M, a Ca^2+^-dependent C-type lectin, has recently been found to correlate with tumourigenesis. This study mainly focused on whether CLEC4M impacts clinical prognosis and how CLEC4M contributes to cisplatin resistance in NSCLC. Our results found that *CLEC4M* was correlated with poor prognosis in patients with lung cancer. In addition, a positive association between *CLEC4M* expression and the IC50 values of cisplatin was found, which suggests that CLEC4M may impact cisplatin sensitivity. In vitro results from cultured A549 and H1299 cells confirmed that CLEC4M could enhance cisplatin resistance, while CLEC4M knockdown led to higher sensitivity to cisplatin in these cells. Further experiments showed that the underlying mechanisms included inhibition of cisplatin-induced cell apoptosis by CLEC4M and improved DNA repair capacity by upregulating XPA and ERCC1 expression. In addition, CLEC4M was able to promote cell migration with or without cisplatin treatment. Collectively, these findings suggest the potential clinical significance of CLEC4M inhibition in overcoming cisplatin resistance in NSCLC patients.

## Introduction

Lung cancer is the leading cause of cancer-related mortality worldwide (GLOBOCAN project of the World Health Organization in 2012, http://globocan.iarc. fr/). Approximately 85% of lung cancer cases are diagnosed as non-small cell lung cancer (NSCLC)^1^ .Platinum-based chemotherapy is the foundation of treatment for most NSCLC patients, including patients with advanced disease, or patients presenting relapse after curative intent surgery^2^, ^3^ . The most commonly used chemotherapy agent is cis-diamine-dichloroplatinum (II), also known as cisplatin, which is used as the backbone of first-line chemotherapy for NSCLC^4^. However, the development of cisplatin resistance remains one of the most challenging problems in the clinical management of patients with NSCLC; cisplatin resistance is a major cause of treatment failure^5^. Accumulating evidence reveals that there are multiple factors involved in cisplatin resistance, including enhanced DNA repair, evasion of apoptosis, drug transport deficiency and reduced drug accumulation^6^-^8^. Although great advances have been achieved in this field, the underlying mechanism of cisplatin resistance remains to be fully explored.

Recently, the role of lectins in tumourigenesis has attracted much attention. Selectins (E-, P- and L-selectin) promote lung metastasis by facilitating the formation of the metastatic microenvironment^9^. Liver sinusoidal endothelial cell lectin (LSECtin), an adhesion molecule, is an important player in colorectal carcinoma liver metastasis^10^. CLEC4M, also known as DC-SIGNR, L-SIGN or CD209L, is a Ca^2+^-dependent C-type lectin. CLEC4M and its homologue DC-SIGN are encoded by the closely related lectin gene cluster on chromosome 19p13.3^11^, ^12^. Both CLEC4M and DC-SIGN are type-II transmembrane proteins that share very similar genetic structures and protein functional domains. The structure of CLEC4M and DC-SIGN consists of an intra-cellular N-terminal domain, a tandem-repeat neck domain and a C-type lectin carbohydrate recognition domain 11, 12. Of note, CLEC4M and DC-SIGNR are expressed in different tissues; CLEC4M is expressed in the endothelial cells of lung, liver and lymph nodes, while DC-SIGN is expressed in the dendritic cells (DCs)^11^. Both CLEC4M and DC-SIGN have long been considered to perform the same function of recognizing a range of pathogens and mediating the endocytosis of ligands^11^, ^12^, but emerging evidence has demonstrated some differences between their roles in immunity and tumour progression.

As a receptor for viruses (HIV, HCV, SARS-CoV, M. tuberculosis and influenza A viruses)^11^, ^12^, CLEC4M has also been recently identified to associate with tumorigenesis. The level of serum CLEC4M was higher in patients with colon cancer than in healthy controls^13^. Subsequent studies confirmed that CLEC4M could promote colon cancer liver metastasis^14^. Studies in gastric cancer mirrored this finding; the serum CLEC4M level is increased in patients with gastric cancer, especially in patients with liver metastasis, and CLEC4M levels strongly correlate with advanced pathological stage^15^. These findings indicate a novel role for DC-SIGNR in tumour metastasis. In the case of lung cancer, similar results were observed; patients with metastasis had a higher level of serum CLEC4M, while patients without metastasis showed lower serum expression of CLEC4M, compared with healthy controls^16^. Based on these observations, one intriguing issue is whether and how CLEC4M contributes to the aetiology of lung cancer and impacts cisplatin sensitivity. This study mainly focused on the association of CLEC4M with clinical prognosis and the particular role of CLEC4M in cisplatin resistance in lung cancer patients.

## Materials and Methods

### GDSC Cell lines

The whole genome expression data and natural log transformed IC50 values of cisplatin for cancer cell lines were obtained from the GDSC database (http://www.cancerrxgene.org/). Based on GDSC release 5.0, a total of 107 lung cancer cell lines were selected to analyse the relationship between CLEC4M expression and cisplatin sensitivity in vitro. Whole-genome mRNA expression information was detected by the Affymetrix Human Genome 133A array. The normalisation method was the Affymetrix Micro Array Suite 5.0 algorithm^17^.

### TCGA samples

TCGA lung squamous cell carcinoma (LUSC) was used to test the relationship between CLEC4M expression and patient prognosis. The TCGA-LUSC datasets contained 504 patients. Based on the clinical information, 119 LUSC patients treated with platinum chemotherapy were screened for subsequent analysis. The whole genome expression data of these datasets were generated by RNA-seq and obtained. The normalized FPKM data and clinical information were downloaded from the NCI genomic data commons (GDC).

### Prognosis analysis in Kmplot

Kmplot software was used to assess the prognostic value of biomarkers^18^. It incorporates the survival information and transcriptome data (obtained by Affymetrix microarray) from the GEO and TCGA databases. A total of 1926 NSCLC patients (133 from TCGA, 1793 from GEO) were included in Kmplot to analysis the association between CLEC4M expression and the overall survival (OS). Of them, 982 NSCLC patients were included to analysis the association between CLEC4M expression and the first progress time (FP).

### Cell lines and cell culture

NSCLC cell lines A549 and H1299 were obtained from the American Type Culture Collection (ATCC). Both A549 and H1299 cells were cultured in RPMI-1640 medium supplemented with 10% foetal bovine serum (FBS, Gibco Company) and 1% penicillin/ streptomycin (Hyclone). These cells were incubated at 37°C in a humidified atmosphere of 5% CO_2_.

### Lentiviruses-mediated CLEC4M knockdown or overexpression

In this study, lentiviruses containing small hairpin RNA (shRNA) specifically targeting human CLEC4M and non-specific shRNA (shControl group), and lentiviruses containing CLEC4M and vector control (vector group) were purchased from Genechem (Shanghai, China). Human CLEC4M shRNA was used to stably silence CLEC4M expression, and lentiviruses containing CLEC4M were used to stably overexpress CLEC4M expression. To silence CLEC4M, 24 h after A549 and H1299 cells were seeded, culture medium mixed with lentiviruses containing CLEC4M shRNA or non-specific shRNA was added into different groups of cells (named shCLEC4M and shControl) for an additional 12 h, and the final multiplicity of infection (MOI) was maintained at 10. Then, supernatants were replaced with normal culture medium, and the cells were continuously cultured for 72 h for subsequent experiments. The transfection process for lentivirus-mediated CLEC4M overexpression is the same as the process of lentivirus-mediated CLEC4M knockdown, which is described in detail above.

### Real-time quantitative PCR analysis (RT-qPCR)

Total RNA was extracted from cells using the TRIzol reagent and was reverse transcribed into cDNA with reverse transcription kits according to the manufacturer's protocols (Thermo Fisher Scientific, Waltham, MA, USA). Real-time quantitative PCR (RT-qPCR) was carried out by SYBR Green Master Mix (Bio-Rad, Hercules, CA) in the CFX96 Touch™ Real-Time PCR Detection System (Bio-Rad, Hercules, CA). The cycle conditions were as follows: 40 cycles, denaturation at 95℃ for 5 seconds and amplification at 60℃ for 30 seconds. GAPDH was normalized as an internal control. Gene expression was calculated using the 2^-ΔΔCt^ method.

### Western blot analysis

Cells were harvested and lysed in RIPA cell lysis buffer containing 1% PMSF and 1% phosphatase inhibitor. A BCA protein assay kit (Beyotime Institute of Biotechnology) was used to determine the concentration of proteins extracted from the supernatants of the cell lysates.

A total of 25 μg protein in each lane was separated by SDS-PAGE (8~12% gels) and then transferred to PVDF membranes (Millipore, Bedford, MA, USA). After blocking with 5% skimmed milk for 2 h at room temperature, PVDF membranes were incubated with primary antibodies at 4°C overnight. After washing the incubated membranes three times with TBST (0.05% M Tris-base, 0.5 M NaCl supplemented with 0.1% Tween-20), the membranes were incubated with horseradish peroxidase (HRP)-conjugated secondary antibodies for 2 h at room temperature. After washing the membranes three times with TBST again, blots were visualized with the ECL plus ChemiDoc^TM^ MP Imaging System (Bio-Rad, Hercules, CA, USA). Band intensities were determined using Image Lab software (version 5.1, Bio-Rad). The antibodies used were as follows: β-actin (1:5000; Proteintech), CLEC4M (1:1000; Abcam), Caspase-3 (1:1000; CST), Cleaved Caspase-3 (1:1000; CST), XPA (1:1000; Proteintech), RAD23B (1:1000; Proteintech), XPG (1:1000; Proteintech), and ERCC1 (1:1000; Proteintech).

### Cell viability assay

Cells stably transfected with lentiviruses were reseeded in 96-well plates. After attachment, cisplatin in increasing concentrations (0, 20, 40, 60, 80 and 100 μM) was added into each well. Twenty-four hours later, cell counting kit-8 (CCK-8, Beyotime Institute of Biotechnology) was used to determine the cell viability according to the manufacturer's instructions.

### Apoptosis analysis

After stable knockdown or overexpression of CLEC4M, cells were reseeded in 6-well plates and treated with or without cisplatin (50 μM) for 24 h according to the group assignment. Subsequently, these cells were harvested, centrifuged, washed and resuspended in cold-PBS, then incubated with both Annexin V-FITC and propidium iodide (PI) (BD Biosciences) for 15 min at room temperature in the dark. Cell apoptosis rates were analysed by FC. In addition, total cellular protein extractions were used to measure the expression of caspase-3 and cleaved caspase-3 by Western blot analysis.

### Transwell assay

Cell migration assays were performed by using Transwell migration chambers (8 μm pore size; Corning, USA). Cell suspensions containing 1% FBS were added to the upper chamber, and the lower chamber was filled with 500 μl of culture medium containing 5% FBS. Twenty-four hours later, non-invading cells in the upper chambers were removed, and the invaded cells in the lower chamber were stained with 0.1% hexamethyl pararosaniline. Representative photos were taken using a TE2000 microscope (Nikon Instruments Inc., Japan) (100×) or SMZ1500 stereomicroscope (Nikon Instruments Inc., Japan) (10×).

### Statistical analysis

Means of two continuous normally distributed variables were compared by independent samples Student's t test. Kruskal-Wallis test or Mann-Whitney U test were used to compare the non-normal continuous variables. Linear regression was used to test the relationship between two quantitative characters. Cox regression was utilized to conduct survival analysis. The log rank test was used to compare the Kaplan-Meier survival curves. All the analyses were conducted by the SPSS software (version 23.0, IBM Software Inc., New York, NY, USA). The Kaplan-Meier curves and scatter plots were drawn with GraphPad Prism 5 (GraphPad Software Inc., San Diego, CA, USA). To select the best cut off value, median, tertile and quartile values were used to divide the sample into high expression and low expression groups. A two-tailed *p* value < 0.05 was considered statistically significant.

## Results

### *CLEC4M* was negatively associated with prognosis in lung cancer

**Figure 1:**
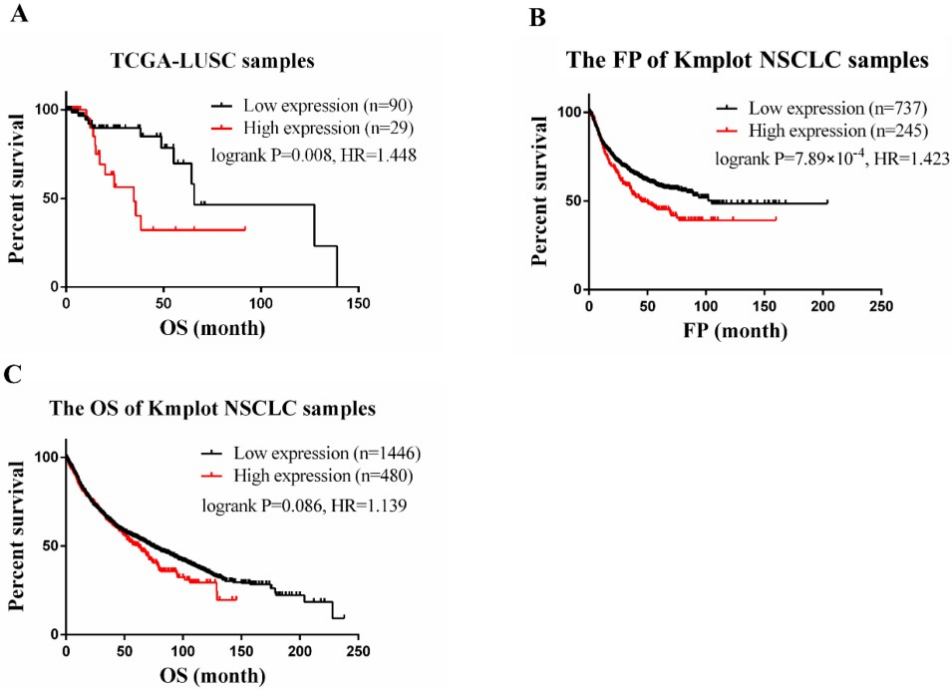
*CLEC4M* expression is associated with poor prognosis in lung cancer. (A) RNA-seq and clinical data of 119 LUSC patients treated with cisplatin chemotherapy were obtained from TCGA. Cox regression was utilized to analyse the correlation between *CLEC4M*expression and OS. (B and C) The correlation of *CLEC4M* expression with OS and FP by analysing 1926 NSCLC patients (133 from TCGA, 1793 from GEO) included in the Kmplot database. Data are presented as the median with interquartile ranges.

In the TCGA-LUSC dataset, after adjusting for age, the expression level of *CLEC4M* was significantly associated with the OS of patients (cox *p*=0.003). To divide the samples into two groups (*CLEC4M* high expression group vs. low expression group), the upper quartile (the best cut-off value) was used. We found that subjects with low expression of *CLEC4M* had longer OS (Log-rank *p*=0.008, HR=1.448, 95% CI: 1.086-1.930). Similar results were found in analysing Kmplot NSCLC samples. Patients with higher expression of *CLEC4M* had shorter OS (Logrank *p*=0.086, HR=1.14, 95% CI: 0.98-1.32) and FP (Logrank *p*=7.89×10^-4^, HR=1.42, 95% CI: 1.16-1.75) than those with lower *CLEC4M* expression. The Kaplan-Meier curves of TCGA-LUSC and Kmplot NSCLC samples are shown in Figure 1 A-C.

### Establishment of cell lines with stable knockdown or overexpression of CLEC4M

**Figure 2:**
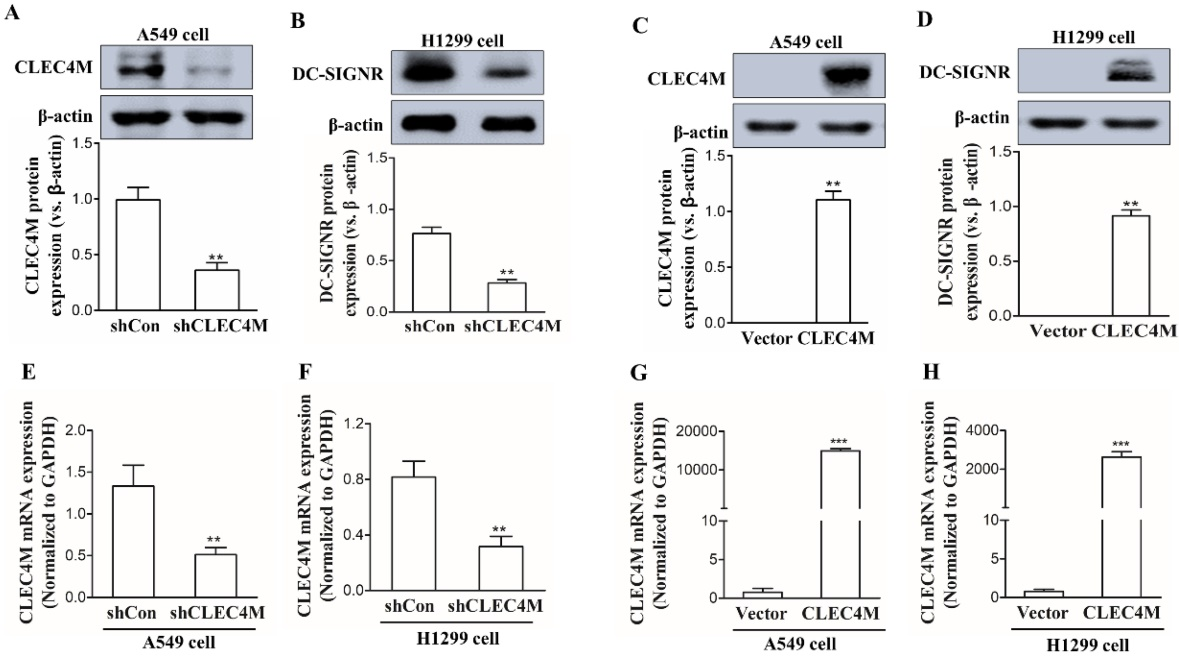
Establishment of A549 and H1299 cell lines with stable knockdown or overexpression of CLEC4M. (A-D) CLEC4M expression was measured after lentiviruses containing shCLEC4M and CLEC4M were transfected into the cells (n=3). (E and H)* CLEC4M* mRNA levels were measured after transfection (n=3). Data are presented as the mean ± SD, ^**^p< 0.01, ^***^p < 0.001 vs. shCon or Vector.

CLEC4M was successfully knocked down in the human lung cancer cell lines A549 and H1299 (Figure 2A, 2B, 2E and 2F); we also observed a remarkable increase in CLEC4M expression at both the mRNA and protein levels, which demonstrated that CLECM was successfully overexpressed (Figure 2C, 2D, 2G and 2H). The stable CLEC4M knockdown or overexpression cell lines were used for subsequent experiments.

### Cisplatin resistance-enhancing effect of CLEC4M in lung cancer cells

**Figure 3:**
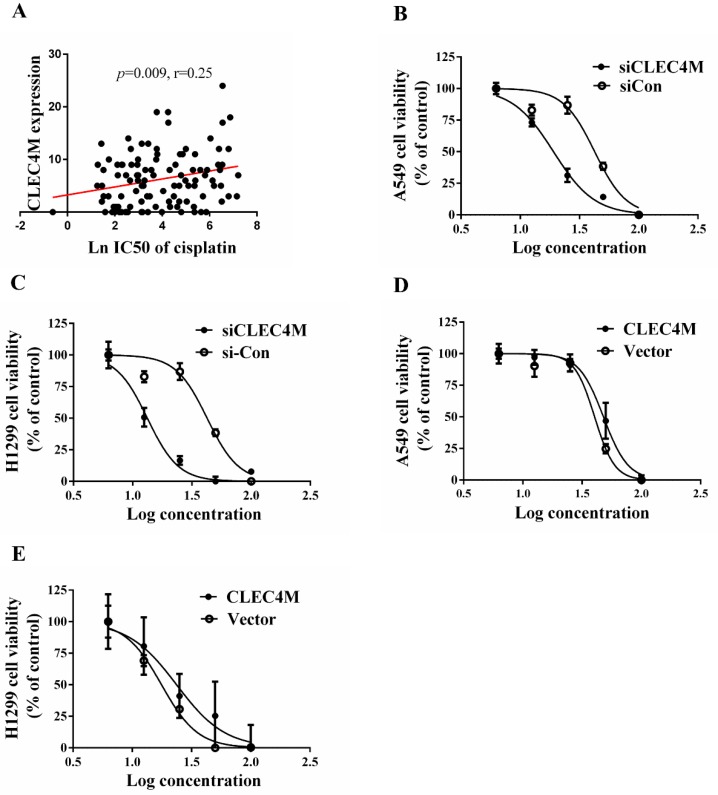
CLEC4M enhances cisplatin resistance in lung cancer cells.(A) The relationship between CLEC4M expression and the IC_50_ value for cisplatin was analysed by linear regression after screening of CLEC4M expression data in 107 lung cell lines obtained from the GDSC database. (B-E) Cell viability was determined by CCK-8 (n=4). Cells transfected with lentiviruses containing shCLEC4M (B and C) or containing CLEC4M (D and E) were reseeded in 96-well plates and treated with increasing concentrations (0, 6.25, 12.5, 25, 50 and 100 μM) of cisplatin for 24 h before measurement. Data are presented as the mean ± SD.

To determine whether CLEC4M influences the sensitivity of lung cancer cell lines to cisplatin, we investigated the impact of CLEC4M on cell proliferation. In the GDSC dataset, the expression of *CLEC4M* was positively correlated with cisplatin IC_50_ values in lung cancer cell lines (*p*=0.009; r=0.250), as shown in Figure 3 A. The detailed information of the lung cancer cell lines is shown in Table S1. In vitro results also showed that cisplatin treatment in combination with CLEC4M knockdown significantly decreased cell viability when compared with cisplatin treatment alone in both A549 (Figure 3B) and H1299 cells (Figure 3C). As the concentration of cisplatin treatment increased, the IC_50_ values for cisplatin in shCLEC4M/A549 cells were decreased, compared with that in shControl/A549 cells. The same findings were observed in shCLEC4M/H1299 cells. In contrast to the CLEC4M knockdown, we observed that CLEC4M overexpression increased cell viability pronouncedly upon cisplatin treatment in both A549 (Figure 3D) and H1299 cells (Figure 3E). The IC_50_ value for cisplatin was higher in CLEC4M/ A549 and CLEC4M/H1299 cells than in vector/A549 and vector/H1299 cells.

### Inhibition of cisplatin-induced cell apoptosis by CLEC4M in lung cancer cells

**Figure 4:**
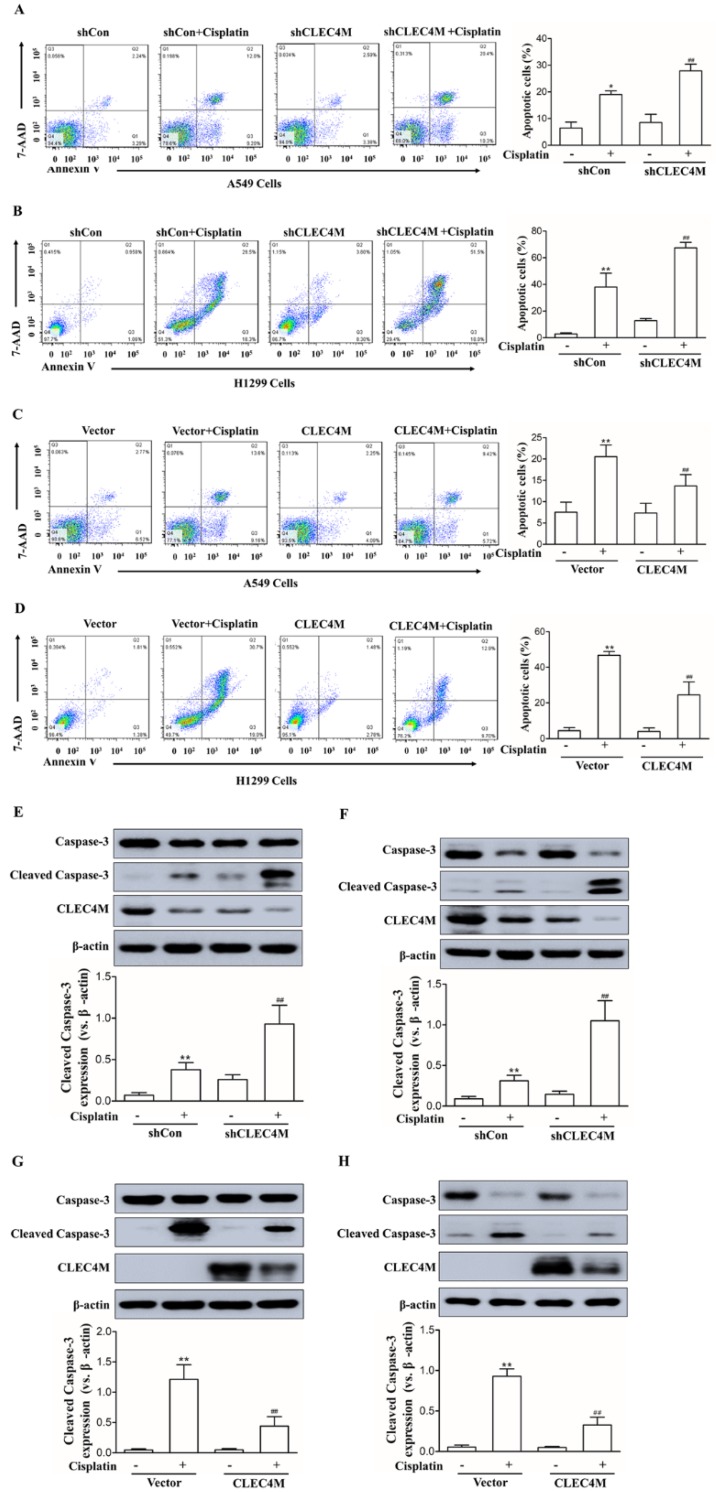
CLEC4M inhibits cisplatin-induced cell apoptosis in A549 and H1299 cells. (A-D) Flow cytometry analysis of apoptotic cells by Annexin V-FITC/PI staining (n=4). Cells with CLEC4M knockdown (A and B) or forced expression of CLEC4M (C and D) were reseeded in six-well plates and treated with or without cisplatin (50 μM) for 24 h before measurement. (E-H) Expression of caspase-3 and cleaved caspase-3 in CLEC4M-silenced A549 and H1299 cells (E and F) or CLEC4M-overexpressing A549 and H1299 cells (G and H) treated with or without cisplatin (50 μM) for 24 h. Data are presented as the mean ± SD,^ *^p< 0.05, ^**^p< 0.01 vs. shCon or vector, ^##^*p*< 0.01 vs. shCon + cisplatin or vector + cisplatin.

The above results showed that CLEC4M enhances cisplatin resistance in lung cancer cells. Next,Annexin V-FITC/PI-stained cells were detected by flow cytometry (FC) to investigate the effect of CLEC4M expression on cell apoptosis induced by cisplatin. As shown in Figure 4A and 4B, cisplatin promoted cell apoptosis across A549 and H1299 cells; CLEC4M knockdown significantly enhanced cisplatin-induced cell apoptosis in A549 and H1299 cells. In contrast, CLEC4M overexpression attenuated cisplatin-induced cell apoptosis both in A549 and H1299 cells (Figure 4C and 4D). Cleaved caspase-3 is a relevant indicator of cell apoptosis. We found that cisplatin treatment led to a significant increase in cleaved caspase-3 expression, and CLEC4M knockdown further upregulated cisplatin-induced cleaved caspase-3 expression (Figure 4E and 4F), but CLEC4M overexpression reduced cisplatin-induced cleaved caspase-3 expression (Figure 4G and 4H).

### Upregulation of XPA and ERCC1 by CLEC4M

**Figure 5:**
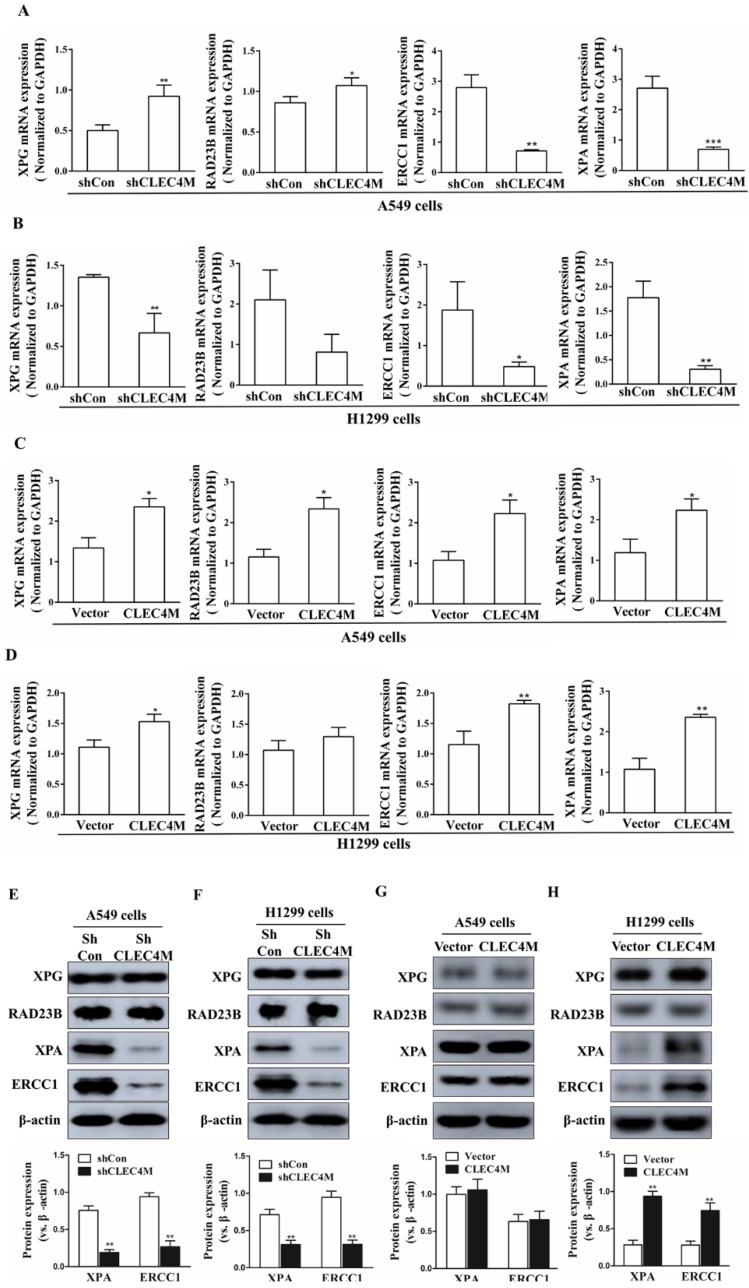
CLEC4M upregulates the protein expression of XPA and ERCC1 in A549 and H1299 cells. (A-D) XPA, RAD23B, ERCC1 and XPA mRNA levels in CLEC4M-silenced A549 and H1299 cells (A and B) or CLEC4M-overexpressing A549 and H1299 cells (C and D) were measured by RT-qPCR. (E-H) XPA, RAD23B, ERCC1 and XPA protein expression in CLEC4M-silenced A549 and H1299 cells (E and F) or CLEC4M-overexpressing A549 and H1299 cells (G and H). Data are presented as the mean ± SD, ^*^p< 0.01, ^**^p< 0.01, ^***^p< 0.01 vs. shCon or vector.

XPG, RAD23B, ERCC1 and XPA are key players in the DNA repair pathway. Our results showed that CLEC4M could regulate ERCC1 and XPA expression in A549 and H1299 cells. CLEC4M knockdown inhibited XPA and ERCC1 mRNA and protein expression (Figure 5A-D). Conversely, overexpression of CLEC4M upregulated the XPA and ERCC1 mRNA levels in these cells, but upregulation of their protein was only observed in H1299 cells (Figure 5E-H).

### Migration-promoting effect of CLEC4M in A549 and H1299 cells

**Figure 6:**
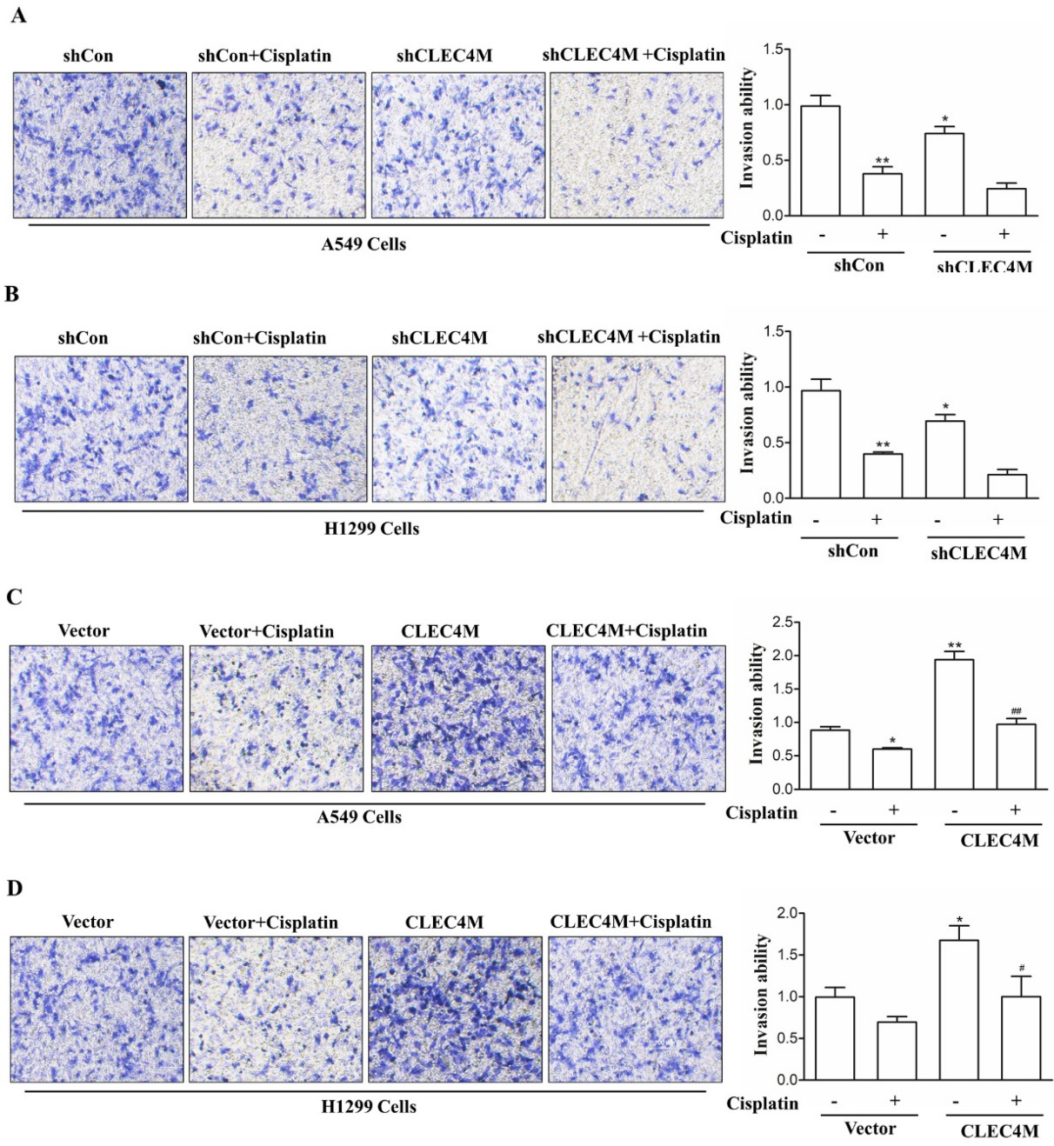
CLEC4M promotes the migration of A549 and H1299 cells. (A and B) The migration ability of CLEC4M knockdown A549 and H1299 cells treated with or without cisplatin (50 μM) for 24 h. (C and D) The migration ability of A549 and H1299 cells with CLEC4M overexpression treated with or without cisplatin (50 μM) for 24 h. Data values are presented as the mean ± SD,^ *^p< 0.05, ^**^p< 0.01 vs. shCon or vector, ^#^*p*< 0.05, ^##^*p*< 0.01 vs. shCon + cisplatin or vector + cisplatin.

In addition, we further detected the effect of CLEC4M on cell migration ability. Consistent with the previous study, we observed that cisplatin (50 μM) decreased the migration of lung cancer cells. Upon cisplatin treatment, A549 and H1299 cells with CLEC4M knockdown showed reduced migration ability when compared with the control groups (Figure 6A and Figure 6B). Conversely, CLEC4M overexpression promoted cell migration and alleviated the inhibitory effect of cisplatin on A549 and H1299 cell migration (Figure 6C and Figure 6D).

## Discussion

Cisplatin resistance is a major obstacle for improving the clinical efficacy of treatment for advanced NSCLC patients. This study mainly investigated the biological effect of CLEC4M on cisplatin resistance. Our results showed that *CLEC4M* was associated with poor patient OS and FP. The positive association between *CLEC4M* expression and the IC50 values of cisplatin suggests that CLEC4M may impact cisplatin sensitivity. *In vitro* results from A549 and H1299 cells confirmed that CLEC4M could enhance cisplatin resistance, while CLEC4M knockdown could significantly increase cisplatin sensitivity. Further experiments showed that inhibition of cisplatin-induced cell apoptosis by CLEC4M and improvement in DNA repair capacity by upregulating XPA and ERCC1 expression were among the underlying mechanisms. In addition, we found that CLEC4M could promote cell migrationwith or without cisplatin treatment.

The C-type lectin CLEC4M is mainly localized on the endothelial cells of liver, lungs and lymph nodes^11^. CLEC4M was previously identified as a receptor to bind and internalize potential ligands, especially viruses 11. Recently, the clinical significance of CLEC4M in cancers has been investigated. The high level of CLEC4M in serum may be a potential molecular marker for the diagnosis of early stage colon cancer^13^. The biological effects of CLEC4M in lung cancer remain unclear. In the current study, prognostic analysis of 119 LUSC patients and 1926 NSCLC patients demonstrated that patients with higher expression of CLEC4M showed poorer OS as well as shorter FP. These results indicate that CLEC4M could lead to worse clinical outcomes in lung cancer patients. Cisplatin resistance is a major reason for the negative prognosis of NSCLC patients. Our study verified the initial speculation that CLEC4M promotes cisplatin resistance; A549 and H1299 cell lines with forced CLEC4M expression had greater IC50 values of cisplatin. These findings indicate that the poor clinical outcome of patients with higher CLEC4M expression may result from enhanced cisplatin resistance.

Cisplatin binds to DNA and forms DNA adducts that will induce DNA damage and tumour cell apoptosis 19, 20. Multiple mechanisms are responsible for cisplatin resistance; one of the most predominant mechanisms is defective apoptosis^6^, ^21^. It is clear that apoptosis defects not only promote carcinogenesis but also lead to chemoresistance^21^. The results of the current study showed that cisplatin significantly induced apoptosis in both NSCLC cells A549 and H1299, while CLEC4M knockdown further enhanced cisplatin-induced apoptosis. Consistently, CLEC4M overexpression inhibited cisplatin-induced apoptosis. These results indicate that CLEC4M suppresses cisplatin-induced apoptosis and thereby leads to cisplatin resistance in NSCLC cells. Apoptotic signals are regulated at several levels. Caspases are essential regulators in the process of apoptosis^22^, ^23^. Of these regulators, caspase-3 is recognized as a key player in controlling cellular apoptosis. Upon receiving signals from the upstream activators (caspase-2, -8, -9 and -10), the inactive precursor of caspase-3 is converted into an active form that is responsible for the majority of cellular destruction^24^. Our results showed that CLEC4M suppressed the upregulation of cleaved caspase-3 that was induced by cisplatin in NSCLC cells; when CLEC4M expression is suppressed, the cisplatin-induced upregulation of cleaved caspase-3 was further increased, suggesting that CLEC4M inhibits cisplatin-induced apoptosis in NSCLC cells, which might be the molecular underpinnings of the role of CLEC4M in cisplatin resistance.

The DNA repair capacity (DRC) of tumour cells has a substantial impact on cisplatin sensitivity and/or resistance^25^, ^26^. Nucleotide excision repair (NER) is considered an important way to sense and respond to cisplatin-induced DNA damage^27^. Four steps are followed to remove DNA lesions by NER: recognition, DNA unwinding, incision by endonucleases, and DNA resynthesize^6^, ^28^. In the early stage, clinical studies revealed that elevated DRC could enhance cisplatin resistance in NSCLC cell lines^29^. A subsequent study also demonstrated that DRC was an independent predictor of clinical outcomes for NSCLC patients treated with cisplatin-based chemotherapies^30^. The components of NER are a series of protein that display distinct functions in DNA repair. ERCC1 is implicated in DNA incision and has received much attention regarding its role in platinum resistance^28^, ^31^. XPA protein binds to the damaged site of DNA and provides docking sites for excision. XPA expression is upregulated in cultured NSCLC cell lines resistant to cisplatin resistance^32^. Our results showed that CLEC4M knockdown inhibited ERCC1 and XPA expression in both A549 and H1299 cells. Conversely, CLEC4M overexpression upregulated ERCC1 and XPA expression in H1299 cells. While the mRNA levels of EXCC1 and XPA were also increased in A549 cells, increases at the protein level were not obvious. However, the reason for the different observations between A549 and H1299 cells is not clear. To some extent, current results suggest that DC-SIGNR may enhance DNA repair by increasing EXCC1 and XPA expression. However, we did not detect the direct DNA repair capacity of CLEC4M, and the molecular action also needs to be elucidated.

Finally, we found that CLEC4M promotes the migration of A549 and H1299 cells with or without cisplatin treatment. Knockdown of CLEC4M expression remarkably inhibited the migration of NSCLC cells, which was in line with the observation that CLEC4M promotes the invasion of colon cancer cells. The role of CLEC4M in tumour metastasis has been reported. For example, CLEC4M promotes colon cancer and gastric cancer liver metastasis^13^, ^14^. In NSCLC patients, the serum levels of CLEC4M were higher in patients with metastasis than in those without metastasis.^16^ Collectively, these findings confirmed a role for CLEC4M in NSCLC migration.

## Conclusion

In conclusion, to the best of our knowledge, this is the first report to demonstrate that higher expression of *CLEC4M* is associated with poor clinical prognosis in lung cancer patients and enhances the resistance of NSCLC cells to cisplatin. Inhibition of CLEC4M expression significantly increased cisplatin sensitivity, suggesting potential clinical significance for targeting CLEC4M in overcoming cisplatin resistance. However, some limitations exist in this study. The underlying molecular mechanisms and signalling pathways of CLEC4M in the regulation of apoptosis and DNA repair remains to be elucidated.

## Supplementary Material

Supplementary table S1.
