# Supplementary material for: CLEC4M is associated with poor prognosis and promotes cisplatin resistance in NSCLC patients
Source: J Cancer. 2019 Oct 19;10(25):6374–83. doi: 10.7150/jca.30139 (PMC6856750; doi:10.7150/jca.30139)
Supplement: Supplementary file 1 — Supplementary table S1. [file jcav10p6374s1.pdf]

CLEC4M is associated with poor prognosis and promotes cisplatin resistance in NSCLC patients

Li-Ming Tan[1,2,6], Xi Li[1,3,4], Cheng-Feng Qiu[2], Tao Zhu[1,3,4], Cheng-Ping Hu[5], Ji-Ye Yin[1,3,4], Wei Zhang[1,3,4], Hong-Hao Zhou[1,3,4], Zhao-Qian Liu[1,3,4*]

Table S1 Detailed information of the lung cancer cell lines

Cell line	Ln IC 50	*CLEC4M* expression (207995_s_at)
IST-SL2	3.406628	13.47463556
KNS-62	2.641739	6.713480964
LCLC-103H	4.719679	5.516910584
LU-134-A	4.750185	11.34806617
LXF-289	3.161664	0.966779717
MS-1	6.547701	24.22563577
NCI-H1304	4.795082	7.501763928
NCI-H1563	4.98031	6.496636393
NCI-H292	5.89784	8.764264349
NCI-H510A	3.250877	6.439159404
PC-14	1.744455	10.91169487
NCI-H226	3.725984	1.927543817
NCI-H23	1.527082	3.883705111
NCI-H460	2.12435	7.597655238
A549	3.631747	7.817825046
COLO-668	4.633801	1.941317557
IA-LM	3.141984	5.224293604
A427	5.858937	0.876468752
NCI-H1651	1.954771	1.076350266
RERF-LC-MS	6.034547	14.27739068
COR-L279	1.429758	13.38729708
LCLC-97TM1	2.986232	7.451941521
NCI-H1975	3.746736	10.4361602
NCI-H810	5.610372	8.694874953
EKVX	6.183365	5.999865978
HOP-62	1.518018	8.65714708
HOP-92	1.255816	5.014155755
ABC-1	1.283161	9.540992841
COR-L105	3.123498	0.693716111
COR-L88	6.511787	2.809866816
IST-MES1	2.981633	10.01725827
DMS-53	6.482946	5.666422918
LU-99A	1.457321	5.564661284
MPP-89	2.191885	0.781196806

MSTO-211H	4.044288	4.531813543
NCI-H520	4.288214	2.687398432
NCI-H596	5.718735	3.550581511
NCI-H441	5.295018	8.152044756
NCI-H2452	5.363383	0.242468815
NCI-H1793	2.951665	9.484382567
NCI-H358	4.506685	5.365016496
NCI-H1155	2.280691	0.389231311
NCI-H28	4.760889	11.72260818
NCI-H1573	4.721239	2.689627541
NCI-H1666	3.47389	3.578084104
NCI-H1703	4.999723	12.48732772
NCI-H1755	5.333582	1.442561069
NCI-H1993	7.209714	7.865103181
NCI-H524	2.139932	1.980992604
SK-LU-1	1.998357	0.55551952
SK-MES-1	4.857482	11.13424906
UMC-11	6.805516	3.011065261
NCI-H838	2.895541	4.205583087
NCI-H1395	3.770087	19.55430059
ChaGo-K-1	2.451125	0.706852196
Calu-3	6.366421	9.450707113
COR-L23	1.728696	3.710556364
LK-2	3.790957	11.93307671
NCI-H1437	3.209855	5.916163089
NCI-H1650	2.943912	7.573977678
NCI-H1693	4.804165	0.725520951
NCI-H1770	6.801738	12.4410431
NCI-H1838	5.639381	0.431911842
NCI-H2126	5.397902	6.860718283
NCI-H2170	3.1356	13.60105882
NCI-H2228	2.110074	9.786993471
NCI-H2342	3.985426	0.40346842
NCI-H2347	5.386718	11.75829188
NCI-H2405	3.124426	2.480581696
NCI-H661	2.047711	1.264195996
DMS-114	2.972983	6.397370201
DMS-273	2.272118	3.69373556
NCI-H1048	2.289497	9.313710221
NCI-H1092	7.15045	3.923209613
NCI-H2029	6.867624	18.01742516
NCI-H209	-0.62197	0.702800606
NCI-H2141	4.489856	3.030008497
NCI-H2227	2.545429	0.827280509

NCI-H345	5.362611	5.845744747
NCI-H446	5.035076	2.388267206
NCI-H526	1.593575	0.661416028
NCI-H82	1.451992	2.049818531
NCI-H2052	5.110637	5.6770381
LU-139	4.560302	9.996804001
SBC-5	4.249885	19.92486011
SBC-1	6.545797	17.24267072
LU-135	2.501227	0.941850384
NCI-H2030	2.473521	7.697403988
NCI-H650	3.398646	0.628991838
NCI-H1299	3.287805	8.122261154
NCI-H2087	2.677199	12.11686955
NCI-H727	6.538156	9.32389853
Calu-6	4.328051	1.270285379
LU-65	5.809373	7.767710758
NCI-H1355	1.891683	0.430864976
NCI-H1792	2.702949	9.590942062
SHP-77	6.399663	12.46136455
NCI-H2009	4.315739	8.624522056
NCI-H2291	6.127042	6.136600098
SW1573	3.768804	12.49614553
SW900	3.365004	3.450853243
BEN	2.321268	9.683874718
CAL-12T	6.43748	8.38748747
CPC-N	3.543486	8.585413642
DMS-79	2.647392	1.158748056
EPLC-272H	2.568121	14.45617965
IST-SL1	4.257404	17.35211457
